# Using 2D-IR Spectroscopy to Measure the Structure, Dynamics, and Intermolecular Interactions of Proteins in H_2_O

**DOI:** 10.1021/acs.accounts.3c00682

**Published:** 2024-02-16

**Authors:** Neil T. Hunt

**Affiliations:** Department of Chemistry and York Biomedical Research Institute, University of York, Heslington, York, YO10 5DD, U.K.

## Abstract

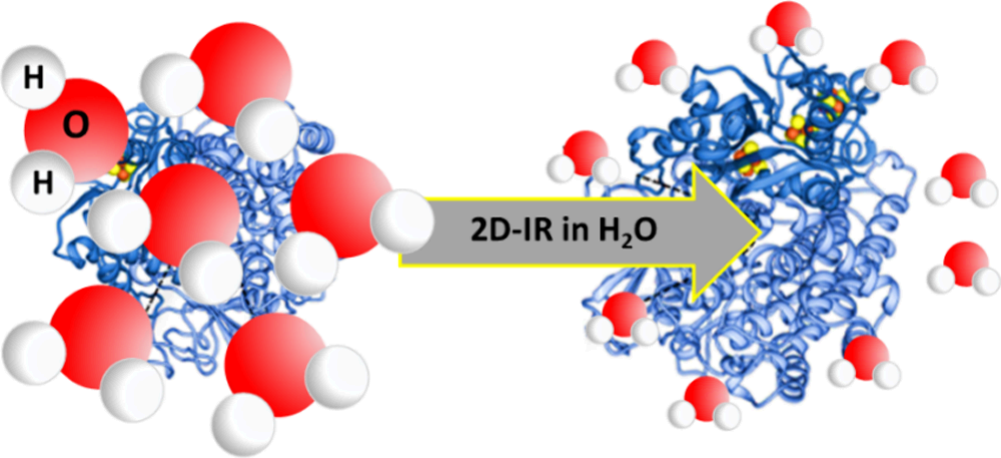

Infrared (IR) spectroscopy probes molecular structure at the level of the chemical bond or functional group. In the case of proteins, the most informative band in the IR spectrum is the amide I band, which arises predominantly from the C=O stretching vibration of the peptide link. The folding of proteins into secondary and tertiary structures leads to vibrational coupling between peptide units, generating specific amide I spectral signatures that provide a fingerprint of the macromolecular conformation. Ultrafast two-dimensional IR (2D-IR) spectroscopy allows the amide I band of a protein to be spread over a second frequency dimension in a way that mirrors 2D-NMR methods. This means that amide I 2D-IR spectroscopy produces a spectral map that is exquisitely sensitive to protein structure and dynamics and so provides detailed insights that cannot be matched by IR absorption spectroscopy. As a result, 2D-IR spectroscopy has emerged as a powerful tool for probing protein structure and dynamics over a broad range of time and length scales in the solution phase at room temperature. However, the protein amide I band coincides with an IR absorption from the bending vibration of water (δ_HOH_), the natural biological solvent. To circumvent this problem, protein IR studies are routinely performed in D_2_O solutions because H/D substitution shifts the solvent bending mode (δ_DOD_) to a lower frequency, revealing the amide I band. While effective, this method raises fundamental questions regarding the impact of the change in solvent mass on the structural or solvation dynamics of the protein and the removal of the energetic resonance between solvent and solute.

In this Account, a series of studies applying 2D-IR to study the spectroscopy and dynamics of proteins in H_2_O-rich solvents is reviewed. A comparison of IR absorption spectroscopy and 2D-IR spectroscopy of protein-containing fluids is used to demonstrate the basis of the approach before a series of applications is presented. These range from measurements of fundamental protein biophysics to recent applications of machine learning to gain insight into protein–drug binding in complex mixtures. An outlook is presented, considering the potential for 2D-IR measurements to contribute to our understanding of protein behavior under near-physiological conditions, along with an evaluation of the obstacles that still need to be overcome.

## Key References

- HumeS.; HithellG.; GreethamG. M.; DonaldsonP. M.; TowrieM.; ParkerA. W.; BakerM. J.; HuntN. T.Measuring proteins in H2O with 2D-IR spectroscopy. Chem Sci.2019, 10, 6448–645631341597 10.1039/c9sc01590fPMC6611063.^1^ Reports the method used to measure 2D-IR spectra of protein amide I band in aqueous solvents.
- HumeS.; GreethamG. M.; DonaldsonP. M.; TowrieM.; ParkerA. W.; BakerM. J.; HuntN. T.2D-Infrared Spectroscopy of Proteins in Water: Using the Solvent Thermal Response as an Internal Standard. Anal. Chem.2020, 92, 3463–346931985198 10.1021/acs.analchem.9b05601PMC7145279.^2^ Describes the use of the water thermal response for normalizing protein 2D-IR spectra in H_2_O.
- RutherfordS. H.; GreethamG. M.; TowrieM.; ParkerA. W.; KharratianS.; KraussT. F.; NordonA.; BakerM. J.; HuntN. T.Detection of paracetamol binding to albumin in blood serum using 2D-IR spectroscopy. Analyst2022, 147, 3464–346935833538 10.1039/d2an00978a.^3^ Reports the measurement of drugs binding to blood serum proteins in biofluid samples using 2D-IR.
- RutherfordS. H.; HutchisonC. D. M.; GreethamG. M.; ParkerA. W.; NordonA.; BakerM. J.; HuntN. T.Optical Screening and Classification of Drug-binding to Proteins in Human Blood Serum. Anal. Chem.2023, 95, 17037–1704537939225 10.1021/acs.analchem.3c03713PMC10666086.^4^ Reports the combination of machine learning with 2D-IR spectroscopy to classify drugs binding to proteins in blood serum samples.

## Introduction

Infrared (IR) spectroscopy has been applied widely to study the structure of proteins. The amide I band reports sensitively on secondary structure because the three-dimensional (3D) spatial arrangement of the backbone leads to vibrational coupling between peptide units that is mediated by hydrogen bonds and through space via electrostatic interactions in addition to that arising directly through the covalent bonding of the backbone.^5^ This coupling and resulting delocalization of the amide I mode generates specific band shapes that are diagnostic of secondary structure elements, such that the amide I band effectively becomes a fingerprint of the protein’s macromolecular structure.^6,7^ The ability of IR spectroscopy methods to measure solution phase samples at ambient temperatures thus allows for the investigation of protein structure under near-physiological conditions.

In IR absorption experiments (otherwise typically referred to as linear IR or Fourier transform IR (FT-IR)), however, the amide I band is typically rather broad and featureless due to the fact that the frequency separations of amide I signatures from the major secondary structure elements (e.g., α-helix, β-sheet, or random coil) are small compared to their line widths, which are inhomogeneously broadened by a mixture of intramolecular dynamics, structural heterogeneity, and solvation effects. The advent of ultrafast two-dimensional IR (2D-IR) spectroscopy has brought new insights into amide I band spectroscopy via the ability to spread the IR spectrum of a molecule over a second frequency axis.^8,9^ The 2D-IR method derives from that of multidimensional nuclear magnetic resonance (NMR) spectroscopies, where a series of laser pulses is used to generate a 2D spectrum in which the fundamental transitions observed in IR absorption spectroscopy appear on the spectrum diagonal, while the off-diagonal region contains peaks that provide information on couplings. The 2D-IR spectrum can thus be thought of as a map of spatial proximity and connectivity of peptide units.^9−11^ In addition, the two-dimensional lineshapes in a 2D-IR spectrum report on structural and solvation dynamics because inhomogeneous and homogeneous broadening effects can be differentiated via the diagonal and antidiagonal line widths, respectively,^12^ while the use of ultrashort laser pulses allows access to further dynamic information such as vibrational relaxation time scales or equilibrium structural fluctuations.

Applications of 2D-IR to study model peptides have shown that distinctive 2D patterns arise from secondary structure elements, such as the “Z-shape” signature of a β-sheet, which derives from off-diagonal peaks linking the characteristic pair of coupled modes near 1620 and 1680 cm^–1^ observed in IR absorption spectra.^6^ These 2D amide I signatures have also been shown to be largely additive, with a study of 16 different proteins showing that 2D-IR spectra can be used to produce a quantitative measure of secondary structure composition, while others have demonstrated comparable accuracy to circular dichroism measurements.^13,14^

Further study of the 2D-IR amide I response has revealed that it has the potential to provide deeper insights. For example, the intensity of the features are also extremely sensitive to the extent of coupling, such that larger, less dynamic or more strongly coupled secondary structure elements produce stronger 2D-IR signals.^15^ This arises from the nonlinear nature of 2D-IR spectroscopy, which reports on changes in the transition dipole moment in a way that is not matched by IR absorption.^13,15,16^ Changes in the size or structural integrity of elements such as an α-helix or β-sheet have also been shown to affect other measurable quantities from a 2D-IR spectrum, such as the anharmonicity of the amide I band (the frequency separation of *v* = 0–1 and *v* = 1–2 transitions).^9,17^ This means that the amide I map potentially reports sensitively the fundamental biophysical parameters of structure, solvation, and dynamics, offering the promise of a label-free measurement that can be linked directly to structure.

Examples of the use of 2D-IR spectroscopy to date include measurements of protein structure in the solution phase,^6^ the time-dependent evolution of conformational change, such as in aggregation and fibril formation,^18^ and the impact of intermolecular interactions, such as ligand binding, on secondary structure and dynamics.^19,20^ 2D-IR has also been used as a structurally sensitive probe of nonequilibrium effects following activation by a temperature jump or photoactivation.^21,22^

## Overcoming the Water Problem

A barrier to performing IR absorption spectroscopy on the amide I band of proteins stems from the H–O–H bending vibrational mode of H_2_O (δ_HOH_). Appearing at 1650 cm^–1^, the δ_HOH_ band overlaps the protein amide I band, leading to the routine use of isotopic substitution of D_2_O for H_2_O in protein solutions; the analogous δ_DOD_ band appears near 1200 cm^–1^, allowing for clear observation of the protein amide I band. H/D substitution has enabled our detailed understanding of the relationship between the amide I band shape and secondary structure. However, the impact of the increased mass of deuterons relative to protons is unclear. D_2_O forms stronger H-bonds than H_2_O by virtue of differences in zero-point vibrational energy.^23^ The kinetic isotope effect shows that the heavier mass can change the reaction rates of enzymes, while the rate of equilibrium structural dynamics, which influences processes such as conformational sampling, will also be affected.^24^ The removal of the energetic resonance between solvent vibrational modes and those of the protein would be expected to alter vibrational relaxation rates, which may impact reaction mechanisms.^25,26^

**Figure 1:**
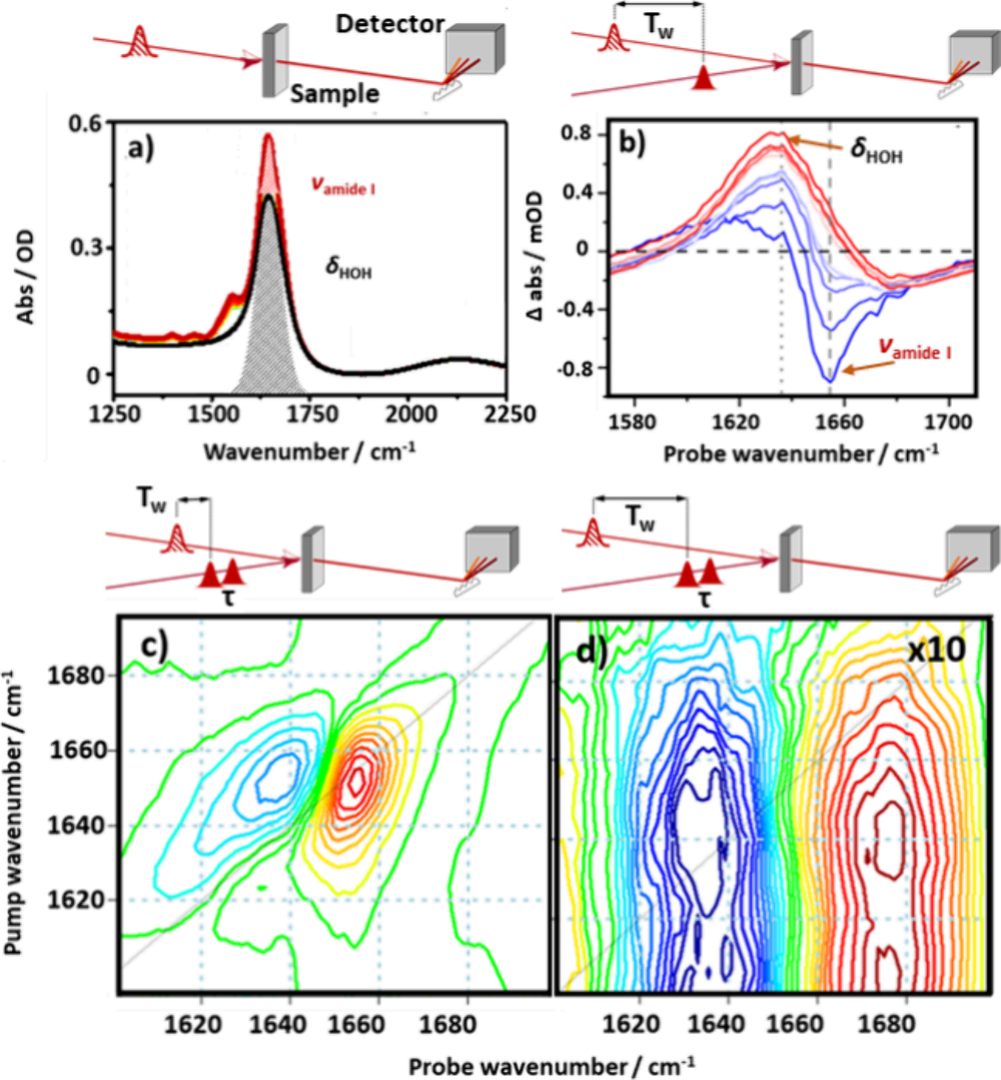
(a) IR absorption spectra of serum albumin solution in H_2_O (red trace) and of H_2_O alone (black trace). (b) IR_pump_-IR_probe_ spectra of serum albumin solution at a range of pump–probe delay times (*T*_w_) from 200 fs (blue) to 10 ps (red). (c, d) 2D-IR spectra of serum albumin solution at two different *T*_w_ values, (c) 250 fs and (d) 5 ps, showing (c) the amide I band and (d) the water thermal response. In each panel, the schematic diagram above the figure shows the experimental method for obtaining the data, where solid pulses indicate “pump” pulses, while hatching indicates probe pulses. Panels (a) and (b) are reproduced with permission from ref (1). Copyright 2019 Royal Society of Chemistry. Panels (c) and (d) are adapted from ref (2). Copyright 2020 American Chemical Society.

It has been shown that 2D-IR spectroscopy can provide the ability to measure protein amide I spectra in H_2_O-rich solutions without the need for isotopic substitution or complex data processing.^1^ The basis of the method can be explained by comparing IR absorption and 2D-IR measurements of a ∼0.5 mM sample of a model protein (serum albumin) in H_2_O (Figure 1). In the IR absorption spectrum, the absorbance near 1650 cm^–1^ arises from two contributions: a large component from the δ_HOH_ band (Figure 1a, black) alongside a smaller one from the protein amide I band (Figure 1a, red). Absorbance is defined by the Beer–Lambert law and scales linearly with concentration and sample path length as well as the extinction coefficient, a molecular parameter for a given vibrational mode that is proportional to the square of the transition dipole moment. It is important to note that, although the absorbance due to water is large, the concentration of H_2_O in the sample is ∼55 M, meaning that the molar extinction coefficient of the δ_HOH_ mode is some 2 orders of magnitude lower than that of the protein amide I mode. Thus, the contribution to the IR spectrum from water arises from a large number of weak absorbers, whereas the reverse is true for the protein contribution.^1^

In a 2D-IR experiment, three laser pulses act on the sample to generate the nonlinear 2D-IR signal (see the experimental diagram in Figure 1c) compared to one in the absorption experiment (Figure 1a). This means that the 2D-IR signal is proportional to the fourth power of the transition dipole moment (the square of the extinction coefficient), and thus, 2D-IR signals from strongly absorbing molecules (proteins) are enhanced relative to those from weak absorbers (water). Thus, the 2D-IR spectrum of the same sample is dominated by the protein amide I band (Figure 1c), with the water response being around 50 times weaker.^1,9,15^

In addition to the different signal intensities, 2D-IR spectroscopy offers the additional ability to separate the water and protein signals via the different vibrational relaxation times of the excited modes. This is exemplified by a series of IR_pump_-IR_probe_ spectra (Figure 1b) obtained at pump–probe delay times (*T*_w_) between 200 fs (blue) and 10 ps (red). At short values of *T*_w,_ the spectrum is dominated by a negative peak at 1650 cm^–1^, arising from the *v* = 0–1 transition of the amide I band, and a positive feature shifted to a lower frequency due to the *v* = 1–2 transition. This amide I signature decays with a characteristic vibrational relaxation time of ∼800 fs.^1^ Although the δ_HOH_ band is excited simultaneously with the amide I band, its response is weaker and vibrational relaxation takes place on time scales of ∼200 fs.^27,28^ This means that, practically, the signal from water does not contribute significantly to the data at early values of *T*_w_, and it decays faster than the protein signal. Figure 1b shows that, at values greater than 2 ps, a broad positive peak appears in the spectrum near 1640 cm^–1^. This is caused by a small change in the δ_HOH_ band of water due to heating following the relaxation of the amide I and δ_HOH_ bands. This small thermal signature persists to microsecond time scales as the sample re-equilibrates.^27^ An important consequence of the fast relaxation of the small initial (negative) water signal being replaced by this (positive) thermal response is that the spectral contribution of water is near-zero at a waiting time of ∼250 fs, allowing the protein signal to be viewed in isolation.^1^ The effect of this spectral evolution on a 2D-IR spectrum is shown in Figure 1c,d. At *T*_w_ = 250 fs (Figure 1c), the amide I band of the protein is visible, while at a value of *T*_w_ = 5 ps, this is replaced by the broad thermal response of the solvent (Figure 1d).^1^

It is important to stress that, although the amide I band of the protein can be measured effectively in the absence of the water response by setting *T*_w_ to 250 fs, the water signal is relatively small and straightforward to account for throughout, meaning that it does not impede measurements at other waiting times, though possible effects of resonance energy transfer from H_2_O may need to be accounted for. Thus, 2D-IR gives access to protein amide I spectroscopy in H_2_O. The only experimental factor that must be considered is the need to limit the sample path length to ∼3 μm in order to restrict the absorbance of the sample to <0.6 at 1650 cm^–1^. Longer path lengths would limit the transmission of pump and probe pulses through the sample and distort or prevent measurement of the 2D-IR signal.^1^ Although this reduction in path length leads to smaller signals relative to measurements in D_2_O, where longer path lengths are routinely used, good-quality spectra can be obtained at submillimolar protein concentrations in H_2_O. Thus, no large increase in protein concentration is required to compensate for the change to H_2_O. However, studies exploiting single isotopically labeled peptides are likely to have to use higher concentrations.

## The Impact of Deuteration on Proteins

**Figure 2:**
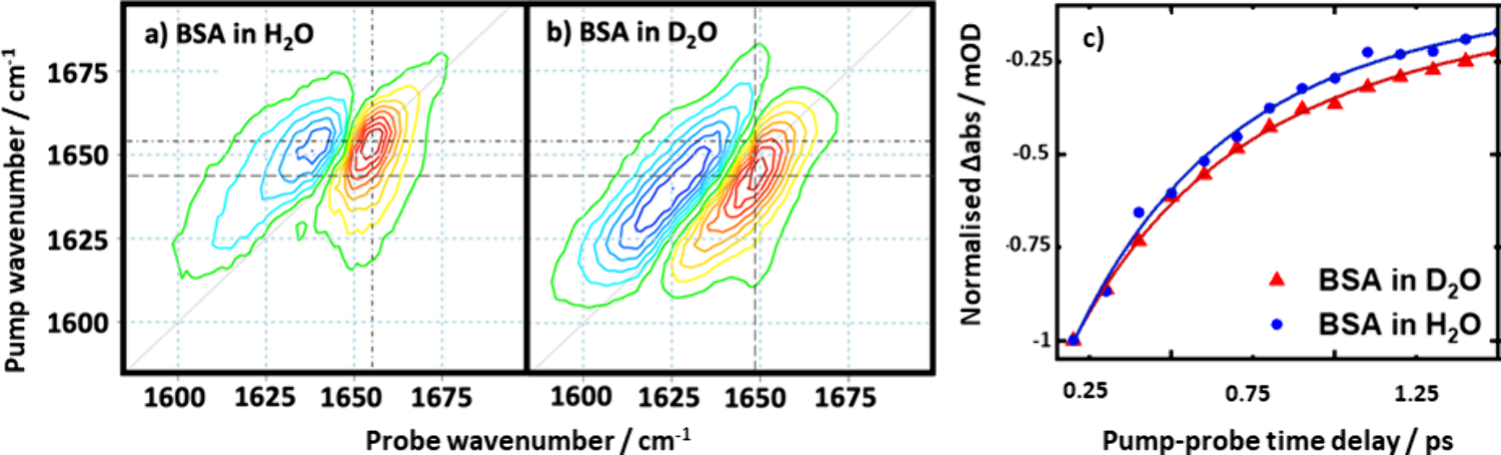
Comparison of 2D-IR spectra of bovine serum albumin (BSA) in (a) H_2_O and (b) D_2_O. (c) Comparison of the vibrational relaxation dynamics of bovine serum albumin in H_2_O (blue) and D_2_O (red).^1^ Panels (a–c) are reproduced with permission from ref (1). Copyright 2019 Royal Society of Chemistry.

A 2D-IR study compared the amide I spectrum of the bovine serum albumin protein (BSA) in H_2_O and D_2_O (Figure 2), showing that the center of the amide I band shifts by around 10 cm^–1^ to a higher frequency in H_2_O,^1^ consistent with experiments using IR absorption and solvent subtraction methods.^29^ There is also evidence for a considerable change in 2D-band shape, with the spectrum in D_2_O appearing more elongated along the spectrum diagonal, while in H_2_O, a narrower line width and a more compact appearance were observed. This data raises a number of interesting fundamental questions: is the shape of a protein 2D-IR amide I band a different in H_2_O? Does this point to changes in structure and/or dynamics occurring upon deuteration? Evidence that would help to answer these questions is currently very sparse, though it has been reported that H/D exchange can lead to varying peptide aggregation pathways, suggesting an impact on fundamental behavior.^30^

The motivation for a detailed and systematic study of the amide I spectroscopy of proteins in H_2_O is clear, and two different approaches to such a problem can be envisioned. One would exploit model peptides and systems with controllable secondary structures.^6−8,31^ However, an alternative approach could harness technological advances, such as rapid-scan 2D-IR,^18^ enabled by pulse shaping technology.^18,32−34^ The ability to study larger numbers of protein samples in practical time scales now means that 2D-IR could follow structural biology approaches in building up libraries of data. Comparing these libraries to large databases of secondary and tertiary structures produced by crystallography or cryo-electron microscopies could allow rapid progress toward predictive tools that can link structure to 2D-IR spectra and vice versa. Such an approach could also be powerful in highlighting how structure and dynamics differ between the crystalline or frozen environment and the solution phase. Here, 2D-IR is equipped to provide insights through the use of transition dipole strength measurements^15,35^ and molecular approaches that exploit changes in coupling between isotopically labeled peptide units to derive information on secondary structure.^36−38^ Of particular value, a recently reported dihedral indexing method is able to identify regions of specific secondary structure via frequency shifts of pairs of coupled peptide units.^37^

The question of how deuteration impacts protein vibrational dynamics is another area where a systematic study could have considerable impact. Preliminary studies have shown a 10% acceleration in the vibrational relaxation of the amide I band of BSA in H_2_O compared to D_2_O.^1^ While this is consistent with the acceleration of vibrational relaxation that would be expected by reinstating the resonance between the δ_HOH_ mode and the amide I mode, it is far less dramatic than that reported in simpler solvent solute systems.^25,26^ Obvious factors that may influence the degree of acceleration include the extent of solvent exposure of individual residues and the relative efficiency of intramolecular vibrational energy transfer. A study of model secondary structures under both solvent conditions would be instructive in this regard.

## Amide I Spectroscopy of Complex Proteinaceous Fluids

In addition to studies of individual proteins in solution, 2D-IR has been applied to measure proteins in blood serum. Serum is the component of blood that remains after the removal of red blood cells, and it is an aqueous mixture of proteins, lipids, carbohydrates, nucleic acids, and minerals. The protein content of human serum is ∼70 mg mL^–1^, composed mainly of albumin (∼35–50 mg mL^–1^) and globulins (∼25–35 mg mL^–1^). The globulin group represents a large number of proteins, dominated by γ-globulins, of which immunoglobulin-G (IgG) is the most abundant (80% of the γ-globulins), followed by IgA (∼13%) and IgM (∼6%).^39^ The concentration ranges of serum proteins extend from albumin, where 35–50 mg mL^–1^ corresponds to 0.5–0.7 mM, to minor constituents, such as IgE, which can be present at nanomolar concentrations. The ability to apply 2D-IR to aqueous solutions opens up two possible new avenues of inquiry: the first is whether 2D-IR can be used as an optical means to detect and quantify proteins within a complex mixture. The protein content of serum provides a molecular snapshot of metabolism and the presence of disease, so 2D-IR amide I spectroscopy in combination with fast scanning technologies could lead to the rapid screening of biofluids for biomedical diagnostics. The second is whether 2D-IR could be used to study protein structure, dynamics, and intermolecular interactions under conditions that are close to physiological conditions or even in vivo. The latter could provide valuable biophysical insights because many biological processes require the formation of large multiprotein clusters or complexes.^40^ 2D-IR, in principle, can measure the spectroscopy of such assemblages but also identify the differences in the structure or dynamics of the component proteins that arise upon interaction, enhancing our understanding of the biophysical factors that control binding.

**Figure 3:**
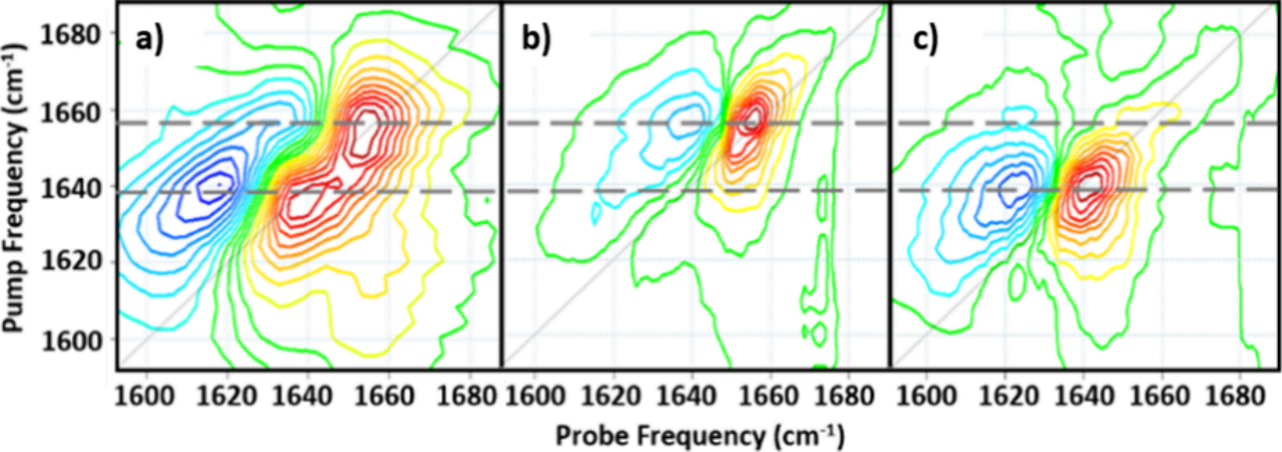
Comparison of the 2D-IR spectrum of (a) blood serum with the 2D-IR spectra of (b) serum albumin and (c) γ-globulin proteins. Gray dashed lines show how the two peaks in the serum spectrum can be assigned to albumin and globulin fractions (see text).^1^ Panels (a–c) are reproduced with permission from ref (1). Copyright 2019 Royal Society of Chemistry.

The application of 2D-IR amide I spectroscopy to unravel protein mixtures was demonstrated in a study that measured the albumin-to-globulin ratio (AGR) of a sample of blood serum.^1^ This seemingly straightforward measurement is a staple of blood tests, but these exploit “wet” chemical methods to derive a differential measurement of albumin and total protein concentration rather than a direct measurement of both fractions. It was demonstrated that the 2D-IR measurement of the amide I band of serum features two peaks on the spectrum diagonal, attributable to serum albumin and globulins, respectively, (Figure 3).^1^ This separation was possible because albumin is primarily α-helical in structure, while globulins are largely derived from β-sheets, the amide I band of which appears at a lower frequency. However, the measurement relies on the superior peak resolution achievable with 2D-IR in comparison to IR absorption. This effect is due to the higher-order dependence upon the transition dipole moment described above,^9^ which narrows the peak widths of the 2D-IR bands relative to IR absorption, reducing overlap. It was demonstrated that the relative peak amplitudes of the albumin and globulin bands, following calibration to account for the different signal intensities per unit concentration of the two components, could be used to determine the AGR, with accuracies (±4%) close to those of wet chemical methods (±1%).^1^

This study was subsequently extended to show that the 2D-IR off-diagonal region could be valuable for identifying changes in other protein constituents^1^ as well as small-molecule components of serum by detecting the presence of glycine via its characteristic 2D peak pattern.^41^ The latter study also allowed a first estimate of the sensitivity limit for 2D-IR measurements of proteins of ∼200 μM. With this sensitivity, 2D-IR could be used to measure the levels of up to 10 proteins found in serum which show concentrations or fluctuation ranges greater than this limit in response to the presence of particular diseases.

Such applications will require continued development of our ability to use 2D-IR fingerprints to unravel complex mixtures, but the use of 2D-IR for biomedical diagnostics could ultimately add a crucial new perspective. While Fourier transform IR (FT-IR) spectroscopy has been used for blood serum studies, such studies generally exploit sample drying to circumvent the water problem, which can introduce artifacts and inaccuracies in concentration measurements. Attenuated total reflectance (ATR) spectroscopy methods and the application of high-brightness quantum cascade lasers do allow measurements in H_2_O, but deconvolution of the δ_HOH_ and amide I bands remains challenging.^42^ Despite this, FT-IR measurements covering the full IR range (400–4000 cm^–1^) have shown promise for disease detection.^43−45^ Recently, a method has used thin samples of serum in transmission to detect the presence of cancers, and it was reported that the basis of the measurement was changes to the amide I region arising from variations in concentration of the 12 most abundant serum proteins.^46^ However, the limited peak resolution of IR absorption and the impact of overlapping water bands prevented the study from identifying the specific proteins or protein combinations responsible and precluded the measurement of protein concentrations. Thus, there is an opportunity for 2D-IR methods to exploit the 2D fingerprint nature of the amide I line shape to deconvolute the protein region of the serum spectrum, while the broader frequency range of FT-IR confers advantages for the rest of the biomolecular fingerprint, which absorb away from regions affected by water.

## Standardization and Quantification Methods

In each of the potential future applications described above involving the use of 2D fingerprints or protein spectral libraries, questions about how to standardize data collection for reliable cross-comparisons will be important considerations. Even with the advantages conferred by pulse shaping, which removes potential issues with correct phasing of the signal, the method of measuring 2D-IR spectra is subject to variables ranging from laser power and beam profile, which can fluctuate within and between measurements, to the quality of the overlap of the pump and probe beams in the sample (Figure 1c,d), which may vary from measurement to measurement. The absolute path length of the sample is also a source of variance between samples, a problem that is exacerbated by the need to produce very thin samples (3 μm), precluding the use of standard spacers between cell windows.

A solution to these problems was found in the thermal response of the water component of the sample.^2^ It was described above that all measurements lead to a small thermal signature from water appearing on picosecond time scales that persists as the sample re-equilibrates relatively slowly by heat diffusion through the cell windows. As this thermal signature arises from the same processes as the 2D-IR protein signal, it depends on the experimental factors outlined above in an identical manner. Inspired by this, it was shown that the magnitude of the thermal signal at *T*_w_ = 5 ps could be used to normalize the protein amide I signature and compensate for the experimental variations between measurements.^2^ A significant advantage of this method is that no additional molecule needs to be added to the sample to act as an internal standard. Tests showed that this water normalization method was robust even when the path length of the sample cell varied by more than 50% and so provides a basis for correction of the signals for experimental factors and a route to accurate production of difference spectra between separate measurements. While it was shown that the approach could be used on two different laser systems,^2^ the question of cross-comparisons between measurements on laser systems still has to be addressed. This is made problematic by variations in laser bandwidth, the selection of optical components in the beam path, and detection systems. However, with the advent of commercial spectrometers and turnkey lasers based on designs for industrial applications where stability and reliability are primary factors, the potential for standardized 2D-IR methodologies becomes an achievable goal.

In addition to enabling reliable comparisons of spectra taken in different experiments, this normalization strategy also provides a route to quantifying concentrations of molecules within mixtures. Measuring the amide I 2D-IR band strength as a function of concentration, with normalization applied in each case, produces a linear calibration plot that can be used as a direct measure of concentration. This needs to be applied with caution in some cases, as changes in secondary structure can have a large impact on the 2D-IR signal strength; however, under conditions where no such change is expected, it can be robustly applied.^47^

Most recently, the application of the water thermal response as an internal, but nonperturbative, standard has been extended to provide a route to preprocessing large 2D-IR data sets prior to screening with multivariate analyses.^47^ The motivation for this work was that the laser systems generally used for high-speed 2D-IR data acquisition have high (100 kHz) pulse repetition rates but relatively low spectral bandwidths (∼80 cm^–1^). The thermal water signal is spectrally broad compared to this, and so, in addition to being used for signal intensity normalization, the shape of the thermal response provided a guide for spectral baseline correction by automatically identifying regions of the signal where the laser intensity was below a threshold value. Normalization and baseline correction were combined with a principal component noise reduction algorithm, and the whole data preprocessing pipeline was shown to improve quantification accuracy and detection limits of 2D-IR in aqueous systems in addition to significantly streamlining the data analysis process.^47^

## Protein–Drug Binding

The binding of drugs to proteins is of interest in areas ranging from fundamental biophysics to the metabolism of drug molecules in the body. Changes to the amide I band of a protein upon drug binding contain information relating to how the secondary structure and local dynamics are modified by the interaction. Applications of 2D-IR amide I spectroscopy in D_2_O have demonstrated significant drug-induced changes, which correlate with function of the drug molecule.^19,48^ These effects were often associated with alterations in the dynamics of the protein rather than large-scale secondary structure changes, and thus, their detection relies on the sensitivity of 2D-IR to factors such as transition dipole moments or anharmonicities, which evade IR absorption measurements.^19,48^ Differences in the H-bonding ability of H_2_O and D_2_O mean that H/D exchange may affect the balance of competitive solvation of the drug molecule and protein binding site, so measurements in the native solvent will be important^49^ while also removing the practical need to exchange buffer solutions or express proteins in deuterated media.

**Figure 4:**
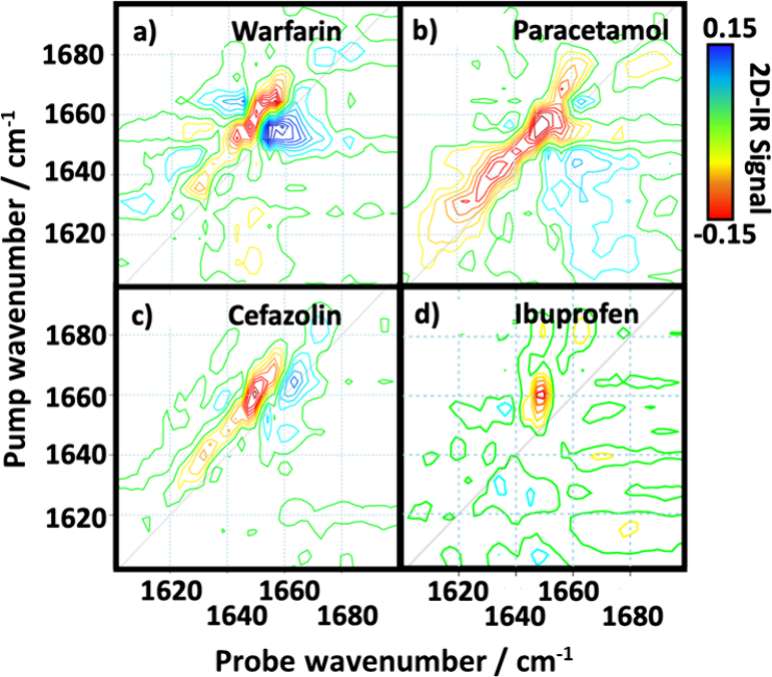
Results of 2D-IR study using machine learning to identify drugs bound to blood serum proteins. (a–d) The spectral changes attributed to each drug by the machine learning algorithm following addition to neat serum: (a) warfarin, (b) paracetamol, (c) cefazolin, and (d) ibuprofen.^4^ Figure adapted from ref (4). Copyright 2023 American Chemical Society.

It has been demonstrated that the amide I band of serum albumin in blood serum is sensitive to drug binding.^3^ Changes in the shape of the band upon the addition of physiological levels of paracetamol (Figure 4) showed not only that drug binding could be detected and quantified, in contrast to IR absorption methods, but also that obtaining physical insight into the interaction was possible at micromolar drug concentrations, well below those detectable directly (the detection of paracetamol binding to albumin at a concentration of 7 μM was reported).^3^ Such insights are valuable because albumin plays an important part in drug delivery. A promiscuous binder of small molecules, albumin interacts with almost all drugs introduced into the bloodstream, influencing distribution and excretion rates and, thus, dosage delivery. Albumin features up to nine binding sites, including two main drug binding sites that are allosterically influenced by seven fatty acid binding sites.^50^ The dependence of albumin binding on the presence of fatty acids means that studies of albumin–drug binding in buffer solution represent a poor model for behavior in vivo because it is virtually impossible to mimic the molecular composition of serum in the laboratory, irrespective of the fact that serum composition also changes dramatically in response to metabolic processes and other bodily functions.^51^ With this in mind, it is exciting that a recent study has shown that the interaction signature of four different drugs binding to serum albumin in human serum is drug-specific, with machine learning able to accurately identify drug-free and drug-containing serum samples from a small training data set.^4^ As a result, the potential now exists for 2D-IR identification, classification, and quantification of bound drugs in a biomedical sample. From a purely biophysical perspective, it also shows that different drugs binding to the same protein produce different changes to the amide I band. This creates significant potential for 2D-IR screening to be applied to measure, understand, and adapt the unique structural and dynamic processes underlying ligand binding, and it invites new studies aimed at understanding the precise nature of the changes for each protein–drug combination so that we can further unravel the information that underlies the 2D-IR plot.

## Future Prospects

The ability to measure the amide I band of proteins in aqueous solution has opened up a number of opportunities in both fundamental and applied aspects of biomolecular spectroscopy. The ability to probe proteins in their natural solvent offers the scope to enhance predictive computational models of structure, dynamics, and intermolecular interactions and adds to our understanding of protein energetics and solvent interactions. Applications in biomedical diagnostics, chemical biology, and drug design are also now feasible.

As 2D-IR technology progresses quickly toward standardized experimental design and as laser technology improves to the point where spectra can be obtained in minutes or less from microliter volume samples, a route from the advanced laser laboratory to analytical technology seems to be emerging. Perhaps the most significant barrier to this progression arises from data analysis, which remains complex and problem-specific. Difficulties in spectral interpretation are exacerbated in label-free applications of 2D-IR because the link between structural change and the amide I band is yet to be fully elucidated. The potential now exists, however, for the interrogation of large 2D-IR data sets with advanced data processing methods, such as the machine learning tools that have been recently demonstrated.^4^ The exciting possibility arises of a predictive tool that is able to mine libraries of protein 2D-IR spectra alongside structural biology databases or artificial intelligence (AI)-based predictions of protein folding to establish spectral signatures of specific structural motifs before offering predictions of spectra of unknown proteins or interpretations of spectral change based on structure. A vitally important contributor to such activity will be establishing a close partnership between 2D-IR spectra and computational simulations in order to bridge the structure–spectroscopy gap.^52^ Molecular dynamics and associated quantum mechanical computations have proved highly effective in interpreting the amide I band in D_2_O. The ability to extend these models into H_2_O would be invaluable.

This Account has focused on label-free studies of the amide I band, which lack the spatial insight from deep within the protein structure obtained by the implantation of labels.^53^ However, these strategies are complementary. Label-free methods are accessible and require no protein modification, which can often be the rate-determining step in many studies employing labels. Label-free methods will thus add a different perspective to that obtained by asking specific local questions with molecular probes and, with continued improvements in comprehension of the spectroscopy–structure relationship, could begin to provide a basis for guiding labeling studies, hence improving efficiency. Ultimately, it is envisaged that both 2D-IR approaches have a role to play in producing a growing toolbox of methodologies for use in a wide variety of applications.
